# Gut microbiota and the early prevention window in type 1 diabetes and latent autoimmune diabetes in adults: a state-of-the-art narrative review on diet and metabolites

**DOI:** 10.3389/fendo.2026.1837746

**Published:** 2026-05-08

**Authors:** Halla Kaminska, Wojciech Wieczorek, Michal Pruc, Monika Janeczko, Zbigniew Siudak, Lukasz Szarpak

**Affiliations:** 1 Department of Children’s Diabetology and Lifestyle Medicine, Faculty of Medical Sciences, Katowice, Medical University of Silesia, Katowice, Poland; 2 Department of Emergency Medicine, Medical University of Warsaw, Warsaw, Poland; 3 Institute of Medical Sciences, The John Paul II Catholic University of Lublin, Lublin, Poland; 4 Institute of Biological Sciences, The John Paul II Catholic University of Lublin, Lublin, Poland; 5 Collegium Medicum, Jan Kochanowski University, Kielce, Poland; 6 Henry JN Taub Department of Emergency Medicine, Baylor College of Medicine, Houston, TX, United States

**Keywords:** early-life exposures, gut microbiome, intestinal barrier, islet autoimmunity, latent autoimmune diabetes in adults, metabolomics, short-chain fatty acids, type 1 diabetes

## Abstract

Autoimmune type 1 diabetes (T1D) is typically the end point of a prolonged process involving genetic susceptibility, the emergence of islet autoimmunity, and progressive loss of pancreatic beta-cell reserve rather than the day hyperglycaemia is first diagnosed. In parallel, research on the gut microbiome has shifted from searching for single causal taxa to examining ecosystem-level functions, microbial metabolites, and host-pathway interactions. In this narrative review, we synthesise prospective, mechanistic, and translational evidence on the role of the gut microbiome in T1D and latent autoimmune diabetes in adults (LADA) using a stage-aware, function-first framework. Across the current literature, the most consistent signals concern impaired intestinal barrier homeostasis, reduced fermentation-related capacity, altered short-chain fatty acid signalling, perturbations in tryptophan-derived and bile acid-related pathways, and their downstream effects on immune regulation and inflammatory tone. In T1D, evidence from prospective early-life cohorts suggests that microbiome maturation, together with diet, infections, and antibiotic exposure, may influence the risk of islet autoimmunity and the tempo of progression toward clinical disease. In LADA, the available evidence is more limited and largely cross-sectional, but supports an immunometabolic interpretation that includes altered microbiome and metabolomic profiles. We propose that the main translational priority is not taxon-specific manipulation, but identification of stage- and age-specific windows of susceptibility across the life course and alignment of these windows with low-risk, mechanistically plausible dietary and microbiome-modulating strategies. This framework may help guide microbiome-informed counselling, study design, and research prioritisation in autoimmune diabetes.

## 1 Introduction

The rising incidence of type 1 diabetes (T1D) across diverse populations at a pace far exceeding what could be accounted for by genetic drift has sharpened attention on environmental determinants of disease risk (1, 2). Among these, the gut microbiota has emerged as a particularly plausible contributor (1–3). It is a rapidly evolving ecosystem, highly responsive to diet, antibiotics, infections, and other early-life exposures, and it produces a dense repertoire of bioactive metabolites that calibrate epithelial integrity and immune development (1, 4, 5). These features position the microbiome not merely as a marker of exposure, but as a potential mechanistic interface through which environmental inputs are translated into immunological phenotypes (1, 2).

Importantly, autoimmune diabetes is not confined to childhood. A substantial proportion of adult-onset diabetes includes an autoimmune component, most commonly captured under the umbrella of latent autoimmune diabetes in adults (LADA), a heterogeneous phenotype that spans a spectrum between classical T1D and type 2 diabetes (T2D) (6, 7). This clinical reality has direct implications for microbiome interpretation: in LADA, autoimmunity unfolds within a more variable metabolic context (adiposity, insulin resistance, medication exposure, and diet patterns) that can independently shape microbial ecology and metabolite outputs (8, 9). Accordingly, any coherent account of microbiome-linked mechanisms in autoimmune diabetes must integrate both staged immune progression and the immunometabolic that is particularly salient in adult-onset disease (7, 8).

T1D itself is increasingly understood as a staged process rather than an abrupt clinical event (1, 10). Long before hyperglycaemia is detected, many individuals traverse a preclinical phase characterised by islet autoantibody seroconversion and progressive dysglycaemia (10, 11). This natural history is essential for framing microbiome research: a biologically meaningful role of the gut ecosystem could manifest either by influencing the initiation of islet autoimmunity (the breaking of tolerance) or by modulating the rate of progression from autoimmunity to overt disease (1–3). In this context, the microbiome is well positioned to shape the immune set point (the threshold at which tolerance to islet antigens fails) through effects on mucosal barrier function, antigen exposure, innate immune tone, and the balance between regulatory and effector T-cell programmes (1, 5, 12).

Mechanistically, several convergent pathways provide a coherent rationale for a microbiome-T1D link (1, 2). First, microbial community outputs influence epithelial barrier integrity and mucosal repair, thereby regulating host exposure to luminal antigens and microbial-associated molecular patterns (1, 4). Second, microbial metabolites (particularly short-chain fatty acids (SCFAs), tryptophan-derived indoles, and bile acid derivatives) signal through host receptors and epigenetic mechanisms to promote immunoregulatory circuits and restrain excessive inflammation (4, 5, 12–14). These signals are closely coupled to the differentiation and stability of regulatory T cells (Treg) as well as the activity of pro-inflammatory effector lineages (including Th17 cells), offering a biologically plausible route by which shifts in microbial function could alter susceptibility to autoimmunity (5, 12–14). Third, early life constitutes a period of heightened plasticity for both the microbiome and the immune system; exposures during this window may have disproportionate and durable effects, which aligns with the timing of islet autoimmunity onset in many at-risk children (3, 10, 15).

Importantly, the most informative evidence for a contributory role of the microbiome currently does not arise from cross-sectional studies performed after diagnosis, where reverse causation and treatment-related confounding (dietary changes, insulin initiation, glycaemic variability, intercurrent infections, and antibiotic exposure) can obscure interpretation (2, 3). Instead, the most informative insights have come from prospective birth and childhood cohorts that collected stool samples and detailed exposure data before autoantibody seroconversion and clinical onset (3, 11, 15). Metagenomic analyses from the TEDDY study suggest that associations with islet autoimmunity and progression to T1D are often more coherent at the level of microbial function than at the level of individual taxa, based mainly on prospective sampling and mechanistic convergence (3, 11). This function-first perspective is conceptually important because taxonomic signatures are frequently inconsistent across cohorts owing to geography, diet, technical pipelines, and host factors, whereas convergent functional perturbations may be more biologically interpretable and potentially more reproducible under current methodological conditions (3, 16). In this review, by a function-first approach we refer to interpreting the gut microbiome primarily through its community-level biological activities and host-relevant outputs - such as fermentation capacity, metabolite production, effects on epithelial barrier homeostasis, and immune-regulatory signalling - rather than through the presence or absence of individual taxa alone.

Nevertheless, several challenges temper causal inference (2, 17). Microbiome composition and function are shaped by a dense network of correlated exposures (feeding practices, infections, antibiotics, socioeconomic context, and household environment), many of which are themselves associated with T1D risk (2, 10, 15). Moreover, microbial communities can shift rapidly over short time scales, while autoimmunity unfolds over years (3, 15). These realities demand careful attention to temporality, repeated sampling, rigorous exposure assessment, and analytical strategies that prioritise mechanistic plausibility over superficial signature hunting. Against this backdrop, the central aim of this review is to synthesise evidence linking microbiome-derived functions (particularly those relevant to barrier integrity and immunometabolism) to the initiation and progression of autoimmune diabetes, with a primary focus on staged T1D and with dedicated consideration of LADA as an adult-onset autoimmune phenotype embedded in a distinct immunometabolic context (1, 18). We also evaluate where diet-based strategies and microbiome-targeted interventions might be most rational across life-course windows of opportunity, while recognising that intervention-level evidence remains limited and is considerably more developed in T1D than in LADA.

## 2 Materials and methods

### 2.1 Study design

This article is a narrative review with a structured literature search and an explicit critical appraisal framework, developed to address the mechanistic complexity and methodological heterogeneity of gut microbiome research in autoimmune diabetes, including T1D and LADA. A formal systematic review and quantitative meta-analysis were not undertaken because the evidence base is highly heterogeneous with respect to populations, microbiome assessment platforms, exposure ascertainment, and stage-specific outcome definitions; pooling such non-equivalent endpoints risks limited biological interpretability. Accordingly, we aimed to provide a critical, stage-aware synthesis with emphasis on functional pathways (metabolite outputs and barrier biology) rather than unstable taxonomic signatures. We prioritised evidence with clear staging and pre-seroconversion sampling to minimise reverse causality.

### 2.2 Literature search strategy and study selection

Structured searches were conducted in PubMed/MEDLINE, Web of Science, and Scopus for publications from 1 January 2009 to 20 February 2026 (final search date). Searches were complemented by manual reference screening of relevant systematic reviews, consensus statements, and landmark primary studies, prioritising prospective birth/childhood cohorts and well-phenotyped adult cohorts addressing LADA. Search concepts spanned four domains: (i) autoimmune diabetes staging and endpoints (autoantibodies/seroconversion, dysglycaemia, clinical onset, β-cell function), (ii) microbiome (16S/shotgun metagenomics; functional pathways), (iii) mechanistic pathways (barrier function, SCFAs, tryptophan-indole-AhR, bile acids/FXR-TGR5, Th17/Treg, innate immune tone), and (iv) exposures/interventions (dietary pattern and fibre, ultra-processed foods, antibiotics,probiotics/prebiotics/synbiotics/postbiotics, early-life exposures). Titles/abstracts were screened for relevance to the prespecified framework and full texts were assessed where needed to confirm eligibility.

Complete database-specific search strings, including Boolean operators and controlled vocabulary where applicable, are provided in **Supplementary Table S1**. Search results were deduplicated before screening. Titles/abstracts and full texts were screened against prespecified eligibility criteria two reviewers independently, and uncertainties were resolved by consultation with senior author. A simplified study selection flow diagram is provided in **Supplementary Figure 1** to improve transparency of the narrative selection process.

### 2.3 Eligibility criteria

English-language, peer-reviewed full-text publications were considered. Conference abstracts without full text were not used to support primary claims. Preprints were not used as primary evidence for key conclusions and, when referenced, were treated as hypothesis-generating. We preferentially included (i) prospective studies with sampling before seroconversion and/or clinical onset, (ii) controlled human studies with clear diabetes phenotyping (including LADA defined by islet autoantibody positivity and adult onset) and transparent microbiome methodology, (iii) studies linking microbiome features to stage-relevant endpoints, and (iv) interventional studies evaluating dietary or microbiome-targeted approaches with metabolic, immunological, barrier-related, and/or clinically staged outcomes. Studies were de-emphasised when diabetes phenotype or microbiome methodology were insufficiently described or when major confounding could not be reasonably addressed.

Studies were not used to support primary mechanistic or translational claims when diabetes phenotyping was unclear, microbiome methodology was insufficiently described, outcome timing could not be aligned to disease stage, or major confounding was judged likely to dominate interpretation. Such studies could still be cited for background context or hypothesis generation, but were weighted lower in the narrative synthesis.

### 2.4 Evidence synthesis and critical appraisal framework

Evidence was synthesised within a stage-aware, function-first framework, mapping findings to disease stages and LADA phenotypic spectra and organising results across convergent mechanistic domains (barrier integrity and antigen/PAMP exposure; microbiota-derived metabolites including SCFAs, indole/AhR, and bile acid signalling; and immune calibration including Th17/Treg balance and innate immune tone). Given the narrative design, no formal risk-of-bias tool was applied; instead, conclusions were weighted by temporality, phenotyping rigour, methodological transparency, and confounder control. When findings were discordant, greater weight was assigned to studies with repeated sampling, detailed exposure capture, multi-omics integration, and/or replication across cohorts, prioritising convergent functional pathways over isolated taxa. Throughout the review, more assertive wording is reserved for claims supported by prospective sampling, stage-relevant phenotyping, convergent mechanistic evidence, and replication or functional consistency across cohorts, whereas more cautious wording is used where evidence is cross-sectional, indirect, or substantially confounded. Key studies underpinning the stage-aware, function-first synthesis are summarised in **Supplementary Table S2**.

## 3 T1D as a staged disease: where the microbiome may shape trajectory and tempo

Contemporary frameworks conceptualise T1D as a staged disease continuum rather than an abrupt clinical event (19–22). Individuals move from genetic susceptibility in the absence of detectable autoimmunity (stage 0), to the appearance of one or more islet autoantibodies with normoglycemia (stage 1), to autoantibodies accompanied by dysglycemia (stage 2), and finally to symptomatic, clinically diagnosable diabetes (stage 3) (19–22). This staging is not simply semantic - it provides a biologically meaningful scaffold for identifying when environmental and microbiome-linked factors might exert leverage, and it clarifies why one-size-fits-all microbiome signatures have been difficult to reproduce across cohorts (2, 3).

From a microbiome perspective, the disease course contains at least two mechanistically distinct bottlenecks (1, 23). The first is the initiation of islet autoimmunity - the point at which immune tolerance to β-cell antigens fails and humoral autoimmunity becomes detectable (1, 11, 24). The second is the rate of progression from established autoimmunity to metabolic decompensation and overt β-cell failure (1, 2, 23). These bottlenecks likely differ in their dominant drivers and thus may be differentially sensitive to microbiome-mediated pathways (1, 2, 11, 25).

At the initiation bottleneck, the microbiome may influence the probability that tolerance is breached by shaping the tone of mucosal immunity and the integrity of the intestinal barrier (1, 3, 11, 16). A microbiome with reduced capacity to support epithelial homeostasis (for example, diminished production of barrier-supportive metabolites) could plausibly increase exposure to luminal antigens and microbial-associated molecular patterns, amplify innate immune activation, and lower the threshold for priming autoreactive responses (1, 4, 11, 16). In this scenario, the gut does not provide a single trigger but rather a permissive inflammatory environment that makes a loss of tolerance more likely in genetically susceptible hosts (1, 16, 24).

At the progression bottleneck, the microbiome may act less as an initiator and more as a disease modifier, shaping the inflammatory environment that determines the pace of β-cell stress and attrition (1, 2, 4, 5). Here, microbiome-driven immunometabolic signals (such as the balance of SCFAs, tryptophan-derived indoles, and bile acid signalling) may affect systemic inflammation, antigen-presenting cell programming, and the stability of regulatory versus effector T-cell circuits (1, 12, 13, 26). Even modest shifts in these pathways could, over time, alter the slope of decline in β-cell function after seroconversion, thereby influencing the latency from stage 1 to stages 2-3 (5, 12, 13).

This distinction has practical implications, based mainly on stage-aware prospective reasoning rather than intervention-level proof (22, 25, 27). If the microbiome primarily affects disease initiation, interventions would likely need to be deployed early, often in infancy or early childhood, during periods of heightened microbial and immune plasticity; however, the relative contribution of initiation versus progression remains unresolved (3, 22, 25). If the microbiome mainly modulates progression, the most relevant window may arise after autoantibody emergence, when slowing the transition to dysglycemia and preserving residual β-cell function become the primary objectives, although evidence for microbiome-directed effects on hard progression endpoints remains limited (22, 27). In reality, both may be true: the microbiome can contribute to the likelihood of entering the autoimmune trajectory and later shape its tempo, but the dominant pathways and optimal intervention targets may differ across stages (1, 2, 25).

Most stage-specific signals reported to date originate from prospective cohorts with repeated sampling prior to seroconversion, whereas post-diagnosis cross-sectional contrasts are more vulnerable to reverse causality from treatment, diet changes, and intercurrent illness.

## 4 LADA: adult-onset autoimmunity in a metabolic context

LADA is phenotypically heterogeneous, occupying a broad spectrum between classical T1D and type 2 diabetes (T2D) (6, 7, 21). In some individuals, LADA resembles T1D: autoimmunity is prominent, C-peptide declines more rapidly, and insulin resistance is relatively modest (7, 28, 29). In others, the clinical picture is closer to T2D, with higher adiposity, greater insulin resistance, and a more overt cardiometabolic risk profile; yet with persistent evidence of islet-directed autoimmunity, most commonly glutamic acid decarboxylase autoantibodies (GADA) (7, 29, 30). This duality is not a mere diagnostic nuance; it has direct implications for how the gut microbiome should be interpreted and how microbiome-targeted strategies might be rationally deployed (8, 28).

Definitions of LADA vary across studies, which can materially influence microbiome associations (6, 31). At minimum, we interpret LADA as adult-onset diabetes with islet autoantibody positivity (most often GADA), but cohorts differ in age thresholds, autoantibody panels and titres, baseline insulin requirement, and the degree of insulin resistance (6, 7, 31). Accordingly, we consider microbiome findings most interpretable when LADA is stratified by (i) autoantibody profile and GADA titre, (ii) BMI and markers of insulin resistance, (iii) C-peptide level and slope of decline, and (iv) time to insulin dependence, as these dimensions capture the immunological vs metabolic balance that likely conditions host-microbe signals (7, 32–34).

Because LADA sits at the intersection of autoimmunity and metabolism, microbiome signals in this condition are unlikely to map neatly onto those described for childhood-onset T1D or for T2D alone (8, 9, 28). Instead, the LADA gut ecosystem may reflect two partially independent axes (8, 29, 35). One axis is primarily immunological, encompassing barrier integrity, mucosal immune tone, and the propensity toward low-grade inflammation - features that plausibly influence antigen exposure and immune activation (8, 28, 35). The other axis is metabolic, shaped by diet quality, adiposity, insulin resistance, and bile acid signalling pathways that can substantially remodel microbial community structure and function (8, 29, 35). Importantly, these axes are not additive in a simple way: metabolic context can amplify or mask immune-associated microbial perturbations, and immune activation can reciprocally influence metabolic regulation and microbial ecology (8, 35).

Consistent with this conceptual model, a clinical multi-omics study has reported distinctive features of the gut microbiota and circulating/faecal metabolome in LADA that associate with autoantibody status, glucose metabolism, and indices of islet function (8). A recurring theme in these analyses is attenuation of microbial functions linked to SCFAs production, suggesting a potential deficit in community-level fermentation capacity and in the generation of metabolites that support epithelial homeostasis and immunoregulation (8, 36). While these findings require replication across ethnically and geographically diverse cohorts and careful adjustment for diet, medication, and adiposity, they reinforce an important point: in LADA, the microbiome should be viewed through an immunometabolic lens, where the same microbial pathway may carry different implications depending on whether autoimmunity, insulin resistance, or both dominate the individual phenotype (8, 9, 28).

In practical terms, this heterogeneity argues against treating LADA as a single entity in microbiome research, particularly because the available evidence remains less mature and more confounded by metabolic overlay than in corresponding T1D studies (6, 9). Stratification by autoantibody profile, C-peptide trajectories, and metabolic status will likely be essential for identifying reproducible functional signatures and for designing interventions aligned with the dominant disease axis - immunological, metabolic, or genuinely hybrid. Yet most translational models remain grounded in staged T1D, and their extension to LADA should therefore be made cautiously (7, 8, 32, 33). In LADA, microbiome interpretation is only as strong as the underlying phenotypic stratification; without separating immune-dominant from metabolic-dominant phenotypes, reproducible functional signals are unlikely to emerge (6, 8, 9).

## 5 Function over taxonomy: a common language for microbiome research

Across cohorts, reported taxonomic differences associated with autoimmune diabetes are often inconsistent (4, 37–41). This variability is unsurprising: microbial community composition is shaped by geography, diet, socioeconomic context, antibiotic exposure, infections, and host genetics, and it is further influenced by technical factors such as sampling protocols, sequencing approaches (16S rRNA profiling versus shotgun metagenomics), and bioinformatic pipelines (4, 37, 38, 40). As a result, ostensibly significant taxa can emerge as cohort-specific artifacts or context-dependent signals rather than generalisable hallmarks of disease biology (38, 40, 41).

For clinical and translational purposes, a more informative and potentially more actionable question is what the ecosystem does, rather than simply which organisms are present, based mainly on prospective and mechanistic convergence rather than on fully resolved reproducibility (1, 4). A function-first lens asks whether the community has sufficient capacity to (i) ferment dietary substrates into SCFAs, (ii) support epithelial integrity and mucosal repair, (iii) shape the host bile acid pool and downstream FXR/TGR5 signalling, (iv) channel tryptophan metabolism toward indole/AhR-relevant outputs, and (v) modulate the intensity and quality of innate immune stimulation within the intestinal lumen (1, 4, 5, 42–47). These functional dimensions map directly onto plausible host pathways - barrier function, immunoregulation, and immunometabolism - that may influence both the initiation and progression of islet autoimmunity (1, 4, 5, 48).

This shift in emphasis has two major advantages. First, it reduces the risk of over-interpreting fragile microbial signatures that fail to replicate because they reflect local ecology or analytic idiosyncrasies rather than shared pathophysiology (38, 40, 41). Second, it provides a practical framework for intervention design, although replication and intervention-level evidence remain limited (1, 5, 42, 43, 46). Many clinically feasible strategies (dietary fibre optimisation, reduction of ultra-processed foods, targeted prebiotics, or metabolite-oriented approaches) aim to reshape community outputs rather than to reconstruct specific strains with high precision (5, 42, 43, 46). In this way, functional readouts offer a common language that better bridges epidemiology, mechanistic biology, and pragmatic intervention strategies, without requiring an unrealistic level of taxonomic determinism (1, 4). This does not mean that functional readouts are free from reproducibility problems; rather, they appear more biologically interpretable than isolated taxonomic signals under current methodological conditions. In microbiome studies of autoimmune diabetes, reproducible signals may be more likely to emerge from convergent functional readouts than from any single taxonomic signature, based mainly on prospective and mechanistic convergence (1, 4).

## 6 The intestinal barrier: a critical interface that is difficult to measure

Intestinal barrier function provides a mechanistic link between diet, microbial metabolites, and immune activation (1, 4, 16). When epithelial integrity is compromised, luminal antigens and microbial-associated molecular patterns (PAMPs) gain increased access to the mucosal immune system, amplifying innate immune activation and raising the background “inflammatory noise” that conditions adaptive responses (1, 4, 16). In such an environment, tolerance becomes harder to maintain: antigen-presenting cells are more likely to adopt pro-inflammatory programmes, and T-cell polarisation can shift toward effector phenotypes at the expense of regulatory circuits that normally restrain autoimmunity (1, 16).

Barrier dysfunction is therefore an attractive explanatory node in autoimmune diabetes, not least because it may be modifiable through microbiome-dependent metabolites, although direct human evidence remains heterogeneous and often relies on indirect surrogates (1, 4, 5). SCFAs and other microbial products can strengthen tight junctions, support mucus layer integrity, and promote epithelial repair; conversely, diets low in fermentable substrate and high in ultra-processed components may erode these protective mechanisms (4, 5, 42). The barrier thus sits at the intersection of inputs (diet and exposures), processors (microbial community function), and outputs (immune tone and antigen handling), making it a mechanistically coherent target in models of islet autoimmunity, although replication across prospective human studies remains limited (1, 4).

At the same time, clinical interpretation demands caution. Many commonly used biomarkers of permeability and barrier disruption are assay-dependent, sensitive to pre-analytical variables, and imperfect proxies for complex, region-specific epithelial physiology. Single-marker approaches therefore risk overstatement - particularly in cross-sectional settings where inflammation, infection, medication use, and glycaemic variability may independently influence barrier-associated readouts (38). However, barrier involvement should not be inferred from single permeability surrogates alone, particularly in cross-sectional settings. The most credible inferences emerge when barrier signals are triangulated across complementary domains: permeability-related markers considered alongside metabolomic profiles (for example, SCFA- and bile acid-linked signatures), indicators of microbial translocation, and systemic or mucosal inflammatory phenotypes (4, 8, 45).

In human T1D studies, signals consistent with altered permeability and barrier disruption have been reported, but the direction and magnitude vary across assays and biomarker panels; moreover, prospective pre-seroconversion assessments remain comparatively scarce relative to cross-sectional studies conducted at or after diagnosis.

Practically, this yields an important methodological and conceptual conclusion: the intestinal barrier is best viewed as part of the mechanistic landscape in autoimmune diabetes, but evidence of barrier involvement is rarely one-dimensional, based mainly on triangulation across prospective and mechanistic evidence rather than on single-marker studies (1). Credible claims require convergent signals across orthogonal measures, careful attention to temporality, and interpretation within the broader immunometabolic context rather than reliance on any single permeability surrogate (4). Metabolite axes appear to provide one of the more coherent mechanistic bridges between diet, microbial ecology, and immune tolerance in autoimmune diabetes, based mainly on prospective and mechanistic convergence (1, 4, 5, 45).

## 7 Microbiota-derived metabolites as an immunometabolic hub

A function-first view of the microbiome naturally converges on its metabolic outputs - small molecules that act as signal carriers between the intestinal lumen, the epithelium, and systemic immunity (4, 5). In autoimmune diabetes, these metabolites are particularly attractive because they offer a mechanistic bridge linking diet (as upstream substrate availability), microbial ecology (as biochemical processing capacity), and immune regulation (as downstream effector programming) (4, 5, 43). Among the best-characterised and more mechanistically coherent metabolite axes are SCFAs, tryptophan-derived indoles signalling through the aryl hydrocarbon receptor (AhR), and microbially modified bile acids that engage FXR/TGR5 pathways (5, 49–51).

### 7.1 SCFAs: the chemical substrate of tolerance and a primary fuel for the epithelium

SCFAs (butyrate, propionate, and acetate) are generated through bacterial fermentation of carbohydrates that escape host digestion, most prominently fermentable fractions of dietary fibre and resistant starch (5, 43). Among these metabolites, butyrate is particularly relevant at the interface of barrier biology and immune regulation (5, 12, 52). It serves as a major energy source for colonocytes, supporting epithelial respiration, mucosal integrity, and repair (5, 52). Simultaneously, it functions as a signalling molecule capable of reshaping transcriptional programmes relevant to tight junction assembly, mucus layer maintenance, and immunoregulatory tone (5, 12, 52).

From an immunological standpoint, SCFAs contribute to the maintenance of tolerance by modulating antigen-presenting cell phenotypes and reinforcing regulatory pathways, including those that support the differentiation and stability of regulatory T cells (Treg) while dampening excessive pro-inflammatory responses (5, 12, 43). In preclinical models of autoimmune diabetes, increasing the availability of acetate and butyrate has been shown to attenuate disease development, at least in part by strengthening tolerogenic mechanisms and constraining inflammatory effector programmes (5, 12). These data do not imply that SCFAs constitute a single missing molecule that prevents T1D; rather, they underscore the broader point that community-level fermentation capacity can influence the host’s baseline immunological set point (5, 43).

The practical implication is comparatively straightforward and clinically reasonable: dietary patterns that promote fermentation and increase SCFA production represent one of the safer and more biologically grounded strategies to support gut-immune homeostasis in populations at increased risk, while replication/interventional evidence for hard autoimmune endpoints remains limited (5, 53). Importantly, this logic does not depend on whether a specific genus rises or falls in a given cohort. What matters is the preservation (or restoration) of function - the ability of the ecosystem to convert dietary substrate into barrier-supportive, immunoregulatory metabolites (4, 5). This should not, however, be interpreted as evidence that increasing SCFA exposure alone prevents seroconversion or delays stage progression in humans.

SCFA-related pathways are among the more mechanistically persuasive links between diet, microbial function, barrier biology, and immune regulation; however, direct human evidence for hard autoimmune diabetes endpoints remains limited. Available human data derive mainly from indirect evidence (metagenomic pathway inference and stool/serum metabolomics) and small mechanistic interventions, while trials powered for hard endpoints such as autoantibody seroconversion or time to clinical onset remain scarce (4, 53).

### 7.2 The tryptophan-indole-AhR axis: mucosal maintenance and repair programmes

A second rapidly expanding area of immunometabolic interest concerns microbial tryptophan metabolism (49, 50). Gut bacteria can convert tryptophan into a range of indole-derived compounds that signal through the AhR in epithelial and immune cells (49, 50, 54). Downstream effects include support for mucosal defence and repair programmes (often discussed in relation to IL-22-associated pathways) alongside broader modulation of effector versus regulatory immune responses (50, 54, 55). Conceptually, this axis is attractive in autoimmune diabetes because it links diet (tryptophan availability, polyphenol-rich foods that shape microbial metabolism, and fibre as a contextual determinant of community ecology) to the quality of mucosal immune regulation (49, 50, 56).

At present, however, the tryptophan-indole-AhR pathway remains more mechanistically persuasive than clinically prescriptive. While the underlying biology provides a coherent framework for understanding how microbial metabolites could stabilise epithelial and immunological homeostasis, direct human interventional data linking modulation of this pathway to meaningful T1D-related endpoints remain sparse (50, 57). As such, the axis is best viewed as a reasonable priority target for biomarker development and mechanistic trials, rather than a basis for guideline-level recommendations (50).

### 7.3 Bile acids and FXR/TGR5 signalling: a gut-metabolism-immunity bridge

Microbial transformations of bile acids generate a signalling landscape that extends well beyond lipid absorption (51, 58). Primary and secondary bile acids act as hormone-like molecules that engage host receptors such as FXR and TGR5, thereby influencing glucose metabolism, energy expenditure, and inflammatory tone (51, 58–60). This axis is especially salient in LADA, where autoimmune β-cell injury frequently coexists with varying degrees of insulin resistance and metabolic dysfunction (51, 58). In this context, diet quality, adiposity, meal timing, and macronutrient composition can reshape bile acid pools; in turn, bile acid signalling may modulate insulin sensitivity, low-grade inflammation, and potentially the broader immunological environment in which islet autoimmunity evolves (51, 58, 60, 61). However, this pathway should currently be interpreted with caution in autoimmune diabetes, as the available evidence remains limited and derives largely from indirect mechanistic data, extrapolation from metabolic disease, and relatively small cross-sectional human studies rather than prospective stage-specific or interventional studies, particularly in LADA.

**Figure 1:**
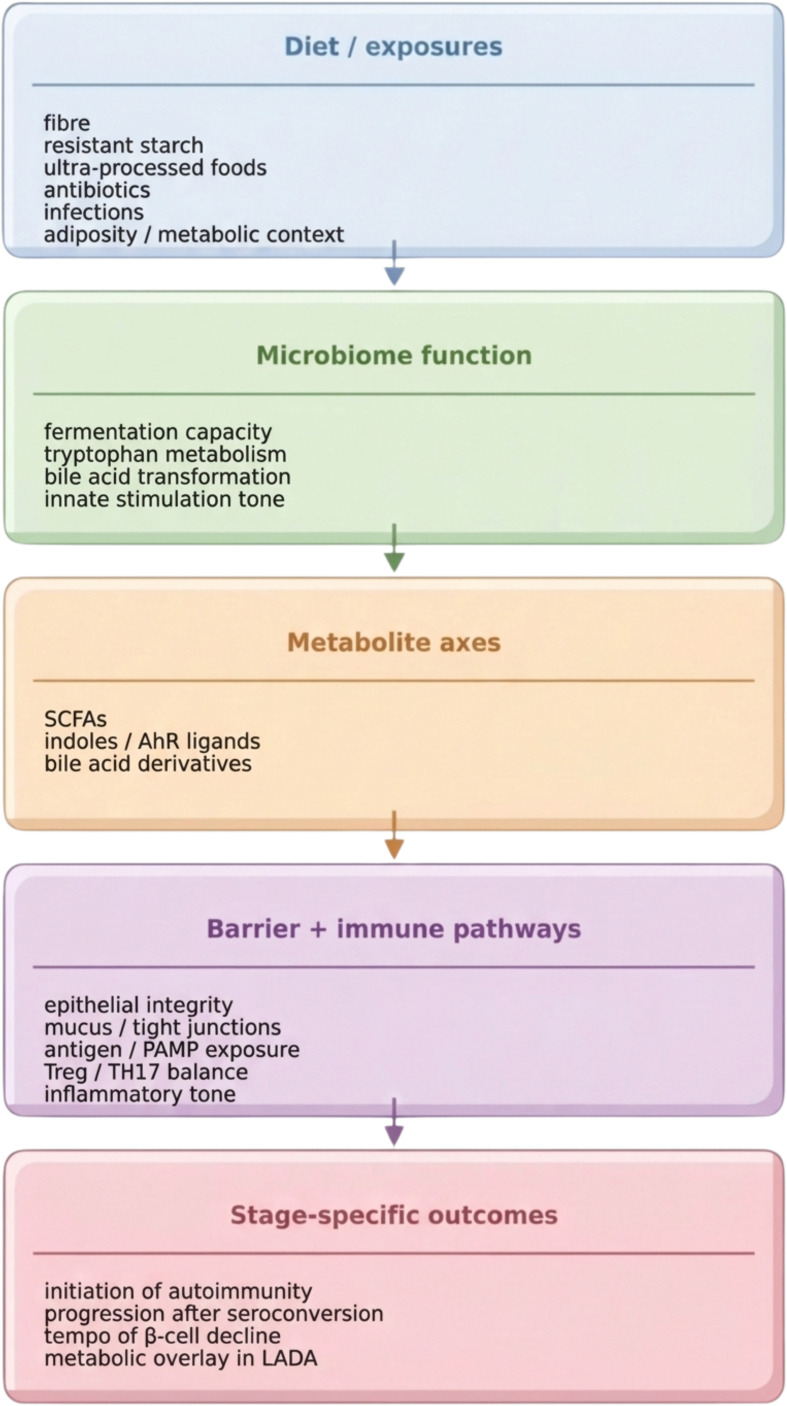
A function-first framework linking diet, microbiome-derived metabolites, barrier biology, and stage-specific outcomes in autoimmune diabetes. Conceptual model illustrating how diet and related exposures shape microbial community function, how this function is expressed through major metabolite axes (SCFAs, tryptophan-derived indoles, and bile acid derivatives), and how these outputs interact with epithelial integrity, antigen handling, and immune regulation. The framework highlights stage-specific consequences, including potential effects on initiation of islet autoimmunity, progression after seroconversion, and the distinct immunometabolic context of LADA.

Although bile acid signalling is most firmly established in metabolic disease biology, it is increasingly relevant to autoimmune diabetes precisely because it embodies the immunometabolic intersection: it provides a mechanistic route by which the same environmental drivers that shape cardiometabolic risk (dietary pattern, body weight, circadian-aligned eating) can also alter host-microbe signalling pathways with immune consequences (51, 58, 62). For LADA in particular, this reinforces the need to interpret microbiome findings through a metabolic lens and to prioritise interventions that address both axes (autoimmune activity and metabolic context) rather than treating them as separable domains, although evidence in LADA remains less mature than in staged T1D (51, 58). The same microbial perturbation can be biologically meaningful or inconsequential depending on timing; accordingly, developmental windows may provide a more informative framework for inference and intervention design than cross-sectional snapshots, based mainly on prospective and mechanistic convergence (5, 12). The main function-first links between diet and related exposures, microbial community outputs, metabolite axes, host barrier/immune pathways, and stage-specific consequences in autoimmune diabetes are summarised in **Figure 1**.

## 8 Windows of susceptibility: when the microbiome is likely to exert maximal leverage

**Figure 2:**
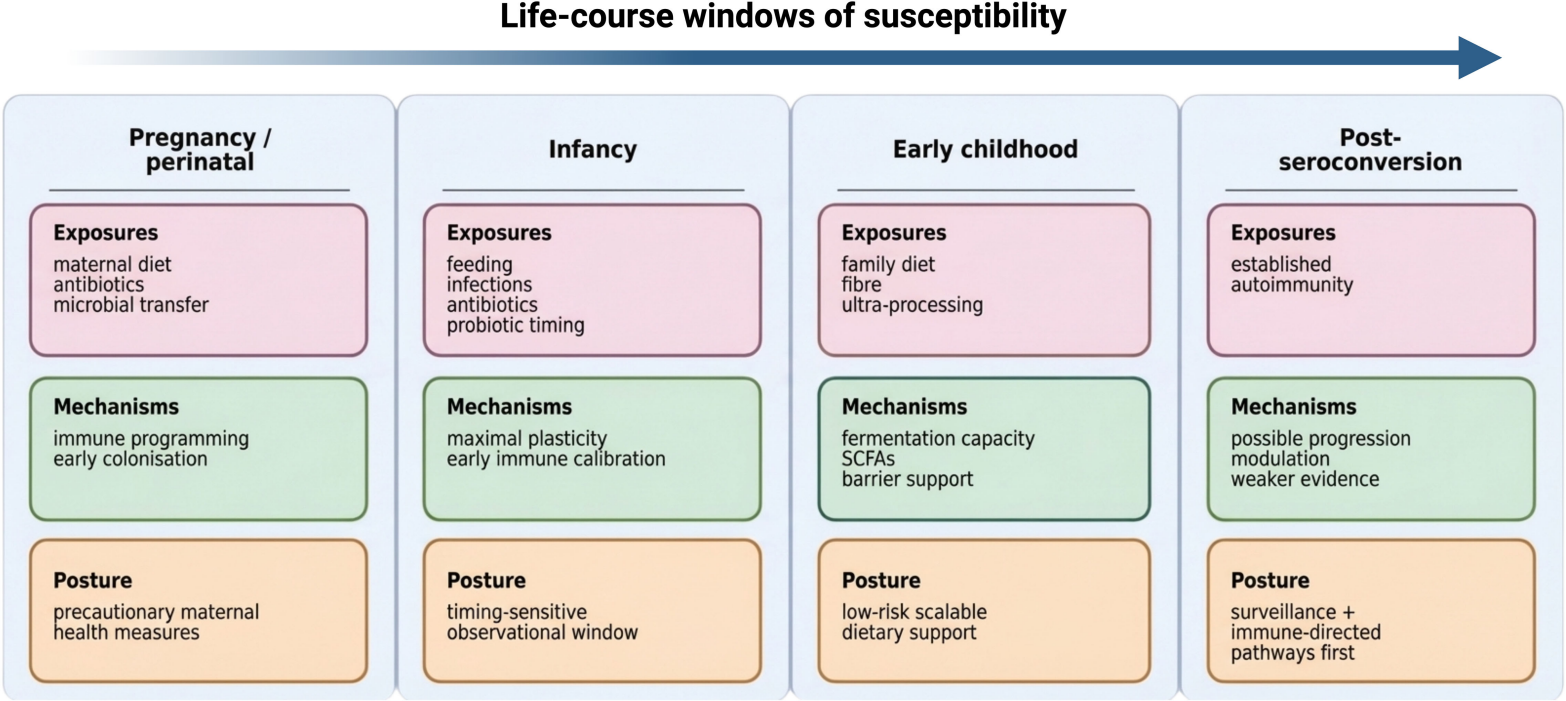
Life-course windows of susceptibility for microbiome-linked influences in autoimmune diabetes. Schematic overview of the major developmental windows discussed in this review, spanning pregnancy/perinatal life, infancy, early childhood, and the post-seroconversion phase. Theimage summarises dominant exposures, putative microbiome-linked mechanisms, and the most realistic translational posture within each window. The model emphasises that biologically plausible leverage and clinically appropriate intervention logic differ across disease stages and across the life course.

One important conceptual advance in the field has been a shift from asking whether the microbiome influences T1D to asking when any influence is most likely to be biologically meaningful and interventionally tractable (15, 63, 64). The gut microbiome at two weeks of age and at twelve years of age are fundamentally different entities: they differ in stability, dominant functional outputs, resilience to perturbation, and responsiveness to dietary and environmental modulation (15, 63, 65). Framing microbiome-T1D research around developmental windows therefore improves causal reasoning (by aligning exposure timing with disease milestones) and clarifies why the same intervention may plausibly help in one period yet be largely inert in another (15, 46, 63). A stage- and age-aware overview of the main windows of susceptibility is shown in **Figure 2**.

### 8.1 Pregnancy and the perinatal period

Pregnancy and the perinatal period represent an upstream window in which immune development is programmed and the ecological conditions for early colonisation are established (63–65). Although the infant microbiome is assembled postnatally, prenatal and perinatal exposures can shape maternal microbial ecosystems, metabolic environment, inflammatory tone, and patterns of early-life microbial transfer (64, 66). For prevention-oriented strategies, this window argues for a precautionary approach focused on a high-quality maternal diet, avoidance of non-essential antibiotics, and caution with extreme elimination diets in the absence of clear clinical indications, while direct intervention-level evidence remains limited (46, 64, 66). The emphasis here is on safeguarding maternal-infant health and minimising avoidable perturbations, rather than pursuing unproven microbiome optimisation protocols (46, 64). A practical near-term implication is to prioritise low-risk maternal health measures especially diet quality and avoidance of non-essential antibiotic exposure while future research should test whether these upstream exposures measurably influence offspring microbiome development and later islet autoimmunity risk.

### 8.2 Infancy (0–12 months): maximal plasticity of the microbiome

Infancy is the period of greatest microbial and immunological plasticity, during which relatively small differences in exposures can yield disproportionate downstream effects (15, 63). Feeding patterns, infections, antibiotic courses, household environment, and the timing and composition of complementary foods collectively shape both community assembly and early functional outputs (15, 65, 67). In this context, observational findings from TEDDY have been particularly provocative: very early probiotic supplementation (within the first 0–27 days of life) was associated with a lower risk of islet autoimmunity among children with the highest genetic risk (68). Importantly, this signal does not constitute proof of causality (confounding by indication and correlated exposures are difficult to exclude in observational settings) and it cannot substitute for randomised interventional evidence (68). Nevertheless, it reinforces the broader biological premise that timing matters, and that infancy may represent a uniquely sensitive window for microbiome-linked modulation of immune trajectories (68, 69). For infants at increased risk, current evidence is more appropriately translated into age-specific feeding principles than into named adult dietary patterns, with emphasis on appropriate early feeding practices, minimally processed complementary foods, and avoidance of unnecessary ultra-processed food exposure. Accordingly, a key research priority is to determine whether precisely timed, low-risk microbiome-modulating interventions in infancy can reproducibly alter trajectories toward islet autoimmunity in genetically susceptible children.

### 8.3 Early childhood: family diet, fermentation capacity, and functional stabilisation

Beyond infancy, the microbiome gradually becomes more stable, and diet begins to dominate its functional architecture (15, 67). The transition toward a family diet changes substrate availability, with direct consequences for fermentative capacity, SCFA production, tryptophan-derived metabolite profiles, and colonisation resistance (3, 15, 46). This period also overlaps with a substantial fraction of islet autoantibody seroconversions in genetically susceptible children, making it highly relevant to both initiation and early progression biology (64, 69).

From a public health standpoint, early childhood likely represents one of the more realistic windows for low-risk, scalable interventions - particularly dietary strategies that support fermentation and barrier biology (for example, fibre-forward patterns and reduced reliance on ultra-processed foods), based mainly on developmental timing and mechanistic convergence (3, 46). Even if such strategies ultimately prove insufficient to prevent T1D outright, they may plausibly improve the gut-immune environment and confer broader health benefits, supporting their use as low-risk, health-promoting approaches while evidence for disease-specific prevention remains limited (46, 66). In practical terms, this supports early-life promotion of a fibre-forward, minimally processed dietary pattern as a low-risk strategy, while future trials should test whether such approaches can modify functional microbiome readouts and stage-relevant autoimmune endpoints.

### 8.4 After seroconversion: secondary prevention and immunological interventions

Once islet autoantibodies are present, the disease process has entered a stage of established autoimmunity (70, 71). The microbiome may still modulate the inflammatory environment and influence the tempo of progression; however, the strongest current interventional evidence at this stage comes from targeted immunological interventions, based mainly on current interventional evidence in staged T1D (27, 70, 71). Consistent with this, in a trial involving high-risk relatives, a single course of teplizumab delayed progression to clinical T1D (71). These data support a stage-specific principle: whereas microbiome- and diet-oriented approaches may be most plausible as upstream modulators (before or around seroconversion), post-seroconversion strategies increasingly require immune-directed therapies if the goal is to alter clinical endpoints meaningfully (27, 70, 71). A key translational question is therefore whether microbiome-targeted strategies can add measurable benefit as adjuncts to structured monitoring and immune-directed secondary-prevention approaches, rather than as stand-alone interventions.

Collectively, a window-based perspective helps integrate microbiome biology with T1D natural history and provides a pragmatic structure for prevention research: intervene early when the system is plastic and tolerance may still be preservable; once autoimmunity is established, prioritise therapies with demonstrated effects on progression while continuing to consider the microbiome as a potential modifier of response and a target for adjunctive, low-risk support (46, 63, 70, 71). Among the low-risk, mechanistically grounded approaches currently most supportable are substrate-based, function-oriented strategies (supporting fermentation and reducing ultra-processing), whereas single-nutrient prevention narratives remain unsupported by trial evidence, particularly in T1D; evidence in LADA remains less mature (46, 72, 73).

## 9 Diet as a tool to modulate the microbiome: what is defensible and what is myth

In the face of population-level variability, one of the more consistently supportable dietary strategies is to support microbial function rather than attempt to correct individual taxa, while recognising that disease-specific interventional evidence remains limited (74–76). In practice, this points to a dietary pattern centred on minimally processed, fibre-rich plant foods that provide fermentable substrates for the gut ecosystem (vegetables, legumes, whole grains, fruits, nuts, and seeds) while limiting ultra-processed foods and sugar-sweetened beverages (74, 77, 78). Such a pattern increases the likelihood of sustained SCFA production, supports epithelial homeostasis, and is typically associated with a lower pro-inflammatory dietary load (74, 76, 78, 79). Importantly, the rationale is function-first and remains mechanistically coherent, based mainly on functional convergence rather than direct proof of T1D prevention, even when taxonomic signatures differ across cohorts, because the target is the community’s metabolic output rather than the presence or absence of any single genus (74–76).

By contrast, attempts to anchor primary prevention of T1D to single, isolated modifications of infant feeding have, to date, proven insufficient (27, 80, 81). In TRIGR, a large randomised clinical trial, replacing conventional cow’s milk-based formula with an extensively hydrolysed formula did not reduce the risk of developing T1D among genetically at-risk children (80). This negative finding is instructive: it cautions against simplistic, single-factor nutritional narratives in a disease process that is multifactorial, staged, and strongly shaped by immune programming and broader environmental context (27, 80).

Fermented foods and probiotic supplements are often presented (especially in popular discourse) as straightforward ways to “repair” the microbiome (82–84). The current evidence does not support such certainty (22, 68, 84). Observational data suggest that very early probiotic exposure may be associated with a reduced risk of islet autoimmunity in selected high-risk infants, but these findings do not constitute a basis for routine probiotic recommendations as a T1D prevention strategy (22, 68). A currently supportable clinical positioning is therefore nuanced: fermented foods may be incorporated as part of an overall high-quality diet when tolerated, and probiotic supplementation may be considered in specific clinical contexts, but any such use should be accompanied by a clear acknowledgement that evidence for T1D prevention remains uncertain and that strain-, dose-, timing-, and host-specific effects are likely to matter (82–85).

Taken together, the dietary message that is most consistent with both mechanistic reasoning and current evidentiary limitations is not that a supplement can “fix” the microbiome, but rather that dietary substrate availability shapes microbial function, and microbial function shapes barrier and immune homeostasis (74–76, 78). This framing helps avoid overclaiming while still providing a rational foundation for low-risk interventions, although replication/interventional evidence for disease-specific prevention remains limited (74, 76).

## 10 Evidence limitations and interpretive pitfalls

Microbiome research in T1D and LADA is constrained by several recurring limitations that complicate causal inference and hamper reproducibility (1, 4, 38, 86). First, much of the literature remains cross-sectional, raising the possibility of reverse causation - particularly after diagnosis, when dietary patterns, medications, glycaemic variability, and intercurrent illnesses can all reshape microbial ecosystems (8, 38, 87). Second, there is substantial methodological heterogeneity, including differences in sampling procedures, sequencing approaches (16S rRNA profiling versus shotgun metagenomics), analytic pipelines, and reference databases, all of which can materially affect reported associations (3, 9, 41). Third, microbiome composition and function are shaped by powerful confounders that are unevenly captured across studies, including antibiotic exposure, infection history, geography, socioeconomic context, household environment, and background diet (3, 16, 39). Finally, studies vary in how they define and time clinical endpoints, spanning islet autoantibody seroconversion, dysglycemia, clinical diagnosis, and the rate of C-peptide decline - outcomes that represent distinct stages of disease biology and may not share the same microbial determinants (3, 11, 38).

In this setting, the more credible conclusions are those built on triangulation rather than single-study signals: convergence between prospective cohort observations (establishing temporality), mechanistic plausibility (linking microbial functions to barrier and immune pathways), and functional consistency across contexts (for example, repeated implication of fermentation capacity and immunometabolic signalling even when taxonomic labels differ) (1–3, 16). This approach reduces susceptibility to signature hunting and better aligns microbiome science with the staged natural history of autoimmune diabetes, which may improve its translational interpretability (1–3). The main human evidence streams discussed in this review are summarised in **Table 1**, organised by phenotype, disease stage, and study design.

**Table 1:** Human evidence map across phenotype, disease stage, and study design in gut microbiome research on autoimmune diabetes.

Phenotype	Stage/clinical context	Evidence type	Most recurrent signal	Interpretive strength	Main limitations	Translational relevance
T1D	Pre-seroconversion/early initiation	Prospective birth and childhood cohorts	Functional shifts outweigh single-taxon signals; recurrent implication of microbiome maturation, fermentation capacity, barrier-related and metabolite-linked domains	Strongest human evidence	Residual confounding; rapid age-related microbiome change; limited causal proof	Best suited to window identification, risk stratification, and upstream prevention hypotheses
T1D	Post-seroconversion/progression before clinical onset	Stage-aware prospective cohorts; limited intervention data	Possible modulation of progression tempo; evidence for microbiome effects on hard progression endpoints remains limited	Moderate	Fewer repeated-sampling studies; hard endpoints uncommon; initiation vs progression difficult to disentangle	Mainly relevant to adjunctive-modulation hypotheses; strongest intervention evidence remains immune-directed
T1D	Stage 3/new-onset or established disease	Cross-sectional, case-control, small mechanistic interventions	Recurrent functional and metabolite-related perturbations; inflammatory and immunometabolic disruption	Supportive, lower-weight	Reverse causation; treatment effects; glycaemic and dietary disruption after diagnosis	Useful for mechanistic inference and human proof-of-concept, not for strong claims about initiation
LADA	Established adult autoimmune diabetes	Cross-sectional, multi-omics, metabolomic-characterisation studies	Immunometabolic pattern; reduced SCFA-related capacity; metabolomic alteration; strong metabolic-context signal	Promising but hypothesis-generating	Heterogeneous case definitions; adiposity/insulin resistance confounding; medication effects; limited replication	Supports immunometabolic framing of LADA and argues against simple extrapolation from childhood T1D
LADA	Prospective/stage-aware evidence	Sparse/very limited	No stable stage-aware signal comparable to pre-seroconversion T1D cohorts	Weakest evidence stream	Lack of prospective cohorts; phenotype dilution; variable autoantibody definitions; metabolic overlay	Major unmet need; priority area for well-phenotyped adult prospective studies

Interpretive strength reflects the review’s stage-aware, function-first synthesis, with greater weight assigned to prospective sampling, stage-relevant phenotyping, repeated measures, convergent mechanistic evidence, and functional consistency across cohorts.

LADA, latent autoimmune diabetes in adults; SCFA, short-chain fatty acid; T1D, type 1 diabetes.

Summary of the main human evidence streams discussed in this review, organised by phenotype (T1D versus LADA), disease stage, and design type (prospective, interventional, or cross-sectional). The table is intended to distinguish where the evidence is strongest, where inference is mainly mechanistic or indirect, and where translational claims should remain cautious.

## 11 Controversies and unresolved questions

Despite substantial progress, several important questions remain unsettled, and these uncertainties should be acknowledged explicitly if the field is to move forward in a clinically useful way. The first concerns causality. At present, the gut microbiome is best understood not as a single upstream cause of autoimmune diabetes, but as a biologically plausible modifier of risk, immune tone, and disease tempo within a host who is already genetically and immunologically susceptible. This distinction matters. It tempers overinterpretation of associative data and helps explain why strong mechanistic plausibility has not yet translated into a simple preventive intervention.

Also unresolved issue is timing. It is still unclear whether microbiome-linked perturbations are most important at the point of islet autoimmunity initiation, during progression after seroconversion, or across both phases through partly different mechanisms. From a clinical perspective, this is not an academic distinction. If the dominant effect lies upstream, then the relevant window is early and developmentally sensitive, often before any autoimmune marker is detectable. If the dominant effect lies downstream, then the microbiome may be more relevant as a modifier of progression than as a determinant of initiation. The current evidence leaves room for both possibilities, but it does not yet define their relative importance with sufficient precision.

Additionally, controversy concerns what should be treated as the most reliable biological signal: taxonomic composition or ecosystem function. Across cohorts, individual taxa have proven difficult to reproduce consistently. By contrast, signals related to fermentation capacity, barrier support, bile acid handling, and tryptophan-derived immunometabolic pathways appear more coherent across settings. Even so, it would be premature to conclude that function has fully solved the reproducibility problem. Functional readouts are more clinically intelligible and mechanistically aligned with host biology, but they too remain dependent on methodology, phenotype definition, and sampling design. The field is therefore moving in the right direction, but it has not yet reached a stable analytic standard.

In LADA, the main unresolved question is how best to separate autoimmune biology from metabolic overlay. This is especially important in adult-onset disease, where adiposity, insulin resistance, diet quality, and medication exposure may all shape microbial structure and metabolite outputs independent of autoimmunity itself. Without careful phenotypic stratification, microbiome findings in LADA risk becoming difficult to interpret and even harder to replicate. In practical terms, studies that do not distinguish immune-dominant from metabolic-dominant phenotypes are unlikely to yield clinically actionable signals.

Finally, there is continuing uncertainty about intervention. A high-quality, fibre-forward, minimally processed dietary pattern is currently supportable as a low-risk clinical approach because it is broadly health-promoting and aligned with the more consistently implicated mechanistic pathways described so far, while evidence that it alters hard autoimmune diabetes endpoints remains limited.

That is very different, however, from claiming that probiotics, supplements, or commercial microbiome products prevent T1D or alter the natural history of LADA. At present, such claims would go beyond the evidence. The field has not yet shown with sufficient consistency that microbiome-directed interventions change hard clinical outcomes such as seroconversion, progression to dysglycaemia, time to stage 3 disease, or preservation of beta-cell function.

Taken together, these controversies should not be viewed as a weakness of the field, but rather as a sign that microbiome research in autoimmune diabetes is entering a more mature phase. A central challenge now is to define when the microbiome matters, through which functions it matters, in which phenotype it matters most, and whether modifying it can change clinically meaningful outcomes.

## 12 Practical implications

### 12.1 Translating a function-first microbiome model into clinical practice

At present, microbiome science in autoimmune diabetes is more useful as a framework for clinical reasoning than as a basis for single, decisive interventions (46, 88). A function-first model centred on SCFAs, indole/AhR signalling, bile-acid pathways, and barrier integrity may help clinicians think in terms of preserving favourable ecosystem outputs and avoiding unnecessary perturbation, while remaining honest about the current limits of prevention evidence (46, 89, 90). In practical terms, this means stage-informed counselling, careful risk communication, and appropriate referral pathways, rather than offering microbiome-focused interventions that imply a level of certainty the field has not yet earned (19, 91, 92).

### 12.2 Risk stratification: match the message to the disease stage

The first practical step is to define the clinical context, because the goals of care differ across stages (19, 91). In stage 0 (genetic or familial risk without autoantibodies), primary prevention remains speculative; the emphasis should therefore be on low-risk, health-promoting exposures and on avoiding harm (19, 46). In stages 1-2 (autoantibodies with or without dysglycaemia), the focus shifts toward secondary prevention: slowing progression, preserving β-cell function where possible, and ensuring structured metabolic follow-up (19, 91, 92). In LADA, care has to integrate immune-aware monitoring with active management of the metabolic context, particularly adiposity and insulin resistance, because these factors influence both disease course and the interpretation of microbiome-related signals (8, 46). This stage-based approach helps avoid two common errors: presenting lifestyle advice as if it could reverse established autoimmunity, or overmedicalising general health advice in individuals who have background risk only (19, 91).

### 12.3 Low-risk, mechanistically grounded actions clinicians can recommend now

Even in the absence of definitive proof that microbiome-targeted strategies prevent T1D, several measures are currently supportable as low-risk, health-promoting approaches because they are broadly beneficial and aligned with the more consistently implicated mechanistic pathways described so far, while evidence that they prevent T1D remains limited (46, 89, 90). One of the clearest low-risk dietary approaches at present is a fibre-forward, minimally processed pattern built around vegetables, legumes, whole grains, fruits, nuts, and seeds (89, 90). The aim is not to prescribe a T1D-prevention diet, but to support fermentation capacity and sustained SCFA generation as an upstream contributor to epithelial integrity and immune homeostasis (89, 93, 94). In the same spirit, limiting ultra-processed foods and sugar-sweetened beverages is a pragmatic way to reduce displacement of fermentable substrates and to lower the overall pro-inflammatory dietary burden (46, 89). Fermented foods may be included as part of an overall high-quality diet when tolerated and culturally appropriate, but they should not be presented as treatment (46). These measures are best framed as general support for gut-immune homeostasis and overall health, not as disease-specific preventive therapy (46, 89).

Beyond diet, antibiotic stewardship, particularly in infancy and early childhood, is best viewed as an important harm-avoidance principle (95–97). The point is not to avoid indicated treatment, but to minimise non-essential antibiotic exposure during sensitive developmental windows when microbiome assembly and immune calibration are especially vulnerable (95, 97). Supplement use should also remain evidence-based. In routine practice, it is more supportable to correct documented deficiencies, including vitamin D or other relevant micronutrients, than to recommend high-dose products marketed on vague claims of microbiome support or immune boosting (46).

Patients often ask whether probiotics or commercial microbiome products can “repair” the microbiome and reduce autoimmune risk. The most accurate answer remains a cautious one. Timing-specific signals have been reported in selected settings, but there is no basis for routine probiotic use as a T1D-prevention strategy (46, 68, 88). Strain-agnostic recommendations should therefore be avoided. If probiotics are used for another clinical reason, it should be made clear that any benefit for islet autoimmunity remains unproven (46, 88).

### 12.4 After seroconversion: prioritise high-leverage pathways

Once islet autoantibodies are present, the patient has entered a stage of established autoimmunity (19, 91). At that point, the most evidence-supported practical step is not a supplement or a microbiome product, but connection to an organised secondary-prevention pathway, based on current intervention-level evidence in staged T1D (19, 91, 92). Clinically, this means referral, where available, to centres or programmes that can provide longitudinal surveillance, including autoantibody follow-up, glycaemic staging, and C-peptide assessment where relevant, together with timely risk communication and evaluation for evidence-based immunomodulatory therapies or clinical trials (19, 91, 98). Diet and lifestyle still matter as supportive modifiers of overall health and, plausibly, inflammatory tone, but they should not be presented as substitutes for monitoring or immune-directed care when the goal is to alter clinically meaningful outcomes (19, 91).

### 12.5 LADA-specific implications: interpret microbiome signals through an immunometabolic lens

In LADA, microbiome-related findings have to be interpreted against considerable phenotype heterogeneity (8, 46). From a clinical standpoint, LADA should be approached as an immunometabolic condition rather than as purely autoimmune diabetes with delayed presentation (8, 46). Where insulin resistance, excess weight, or broader cardiometabolic risk are present, these warrant active management because they influence not only disease progression but also microbial ecology and metabolite output (8, 46). At the same time, β-cell reserve should be monitored and treatment intensified early when progression becomes evident (8). Dietary strategies that improve metabolic health may also support microbiome function, including SCFA-related capacity and bile-acid signalling, which supports a plausible dual-benefit rationale even when diabetes-specific microbiome endpoints remain uncertain, particularly in LADA, where evidence remains less mature and more confounded by metabolic overlay (46, 89, 90). In adults with LADA, particularly when metabolic overlay is prominent, a Mediterranean-style dietary pattern may provide a pragmatic template because it operationalises the core goals of higher fibre intake, lower ultra-processing, and broader cardiometabolic risk reduction; however, this should currently be regarded as a clinically reasoned extrapolation rather than a LADA-specific evidence-based prescription.

### 12.6 A pragmatic clinical message

For now, a supportable near-term translational position is to support microbial function through diet quality and harm-avoidance, interpret microbiome claims conservatively, and ensure that individuals who enter autoimmune stages are linked, where feasible, to structured monitoring and secondary-prevention pathways, while replication/interventional evidence remains limited and is stronger in T1D than in LADA (19, 46, 89, 91). This is a biologically coherent and clinically conservative position that is broadly aligned with the current evidence base (46, 91).

## 13 Future directions and research agenda

To accelerate translation and improve reproducibility, future work should explicitly align microbiome measurements with autoimmune diabetes staging, prioritise functional readouts over taxonomic signatures, and evaluate metabolite-oriented strategies against clinically meaningful endpoints (3, 22, 46). Progress is likely to depend on stage-specific primary outcomes (time-to-seroconversion [stage 0-1], time-to-dysglycaemia [stage 1-2], time-to-clinical onset [stage 2-3], and preservation of the C-peptide slope) embedded within repeated-sampling designs that capture key time-varying exposures (antibiotics, infections, diet, growth trajectories, and medications) to strengthen temporality and reduce residual confounding (3, 22, 41). Multi-omics integration should increasingly be treated as a priority analytic standard, combining shotgun metagenomics with targeted metabolomics (SCFAs, indoles, and bile acids) and immune phenotyping to connect community function to host pathways, alongside harmonised minimal confounder sets and reporting (antibiotic courses, recent infections, dietary pattern assessment, BMI/adiposity, socioeconomic proxies, and geography) to enable cross-cohort comparability (1, 4, 99, 100). Translational efforts should preferentially focus on pragmatic, paediatric-safe interventions that shift microbial function (prebiotics/fibre formulations, postbiotics, and dietary patterns that increase fermentation capacity), particularly in staged T1D (1, 22, 46). In parallel, LADA studies should use transparent case definitions and prespecified stratification by autoantibody profile/titre, insulin resistance, and C-peptide trajectory to avoid phenotype dilution, while recognising that evidence in LADA remains less mature (1, 22, 46).

Unlike staged T1D, LADA does not yet have a validated stage-based framework anchored to preclinical autoimmunity and dysglycaemia. A pragmatic stage-informed model for LADA may therefore need to be constructed around clinically tractable domains rather than formal stages: autoimmune burden (number and titre of islet autoantibodies), residual β-cell reserve (fasting or stimulated C-peptide and its trajectory), metabolic overlay (BMI, insulin resistance, and broader cardiometabolic context), and tempo of progression toward insulin dependence. In translational terms, such a framework could help distinguish immune-dominant from metabolic-dominant phenotypes, improve comparability across cohorts, and better align microbiome-oriented strategies with the dominant disease axis in a given patient. At present, however, this should be considered a working stratification model rather than a formally validated staging system. In addition, microbiome markers should be tested as modifiers of response to immune-directed secondary-prevention therapies rather than positioned as stand-alone treatments, and key claims should be supported by pre-registered analyses and replication across cohorts to curb signature inflation.

Beyond diet- and metabolite-oriented strategies, a further translational frontier is to intervene more directly at the gut-immune interface itself. Potential directions include modulation of gut lymphocyte trafficking or homing, oral antigen-based approaches aimed at promoting mucosal tolerance, and microbiota-transfer strategies such as faecal microbiota transplantation. Conceptually, these approaches are attractive because they more directly target the barrier-mucosal immune axis discussed above; however, in autoimmune diabetes they remain investigational, with limited stage-specific human evidence and unresolved questions regarding timing, patient selection, durability, and safety.

Importantly, gut microbiome- and metabolite-related signals should not be assumed to have the same biological meaning in T1D and LADA. In staged T1D, the dominant interpretive framework is developmental and autoimmune, with greater emphasis on early-life microbiome maturation, barrier biology, and stage-specific progression after seroconversion. In LADA, by contrast, microbiome and metabolite profiles are more likely to be shaped by a hybrid immunometabolic context in which autoimmunity coexists with variable degrees of adiposity, insulin resistance, diet-related exposures, and medication effects. This distinction has translational implications: interventions should not be considered interchangeable across the two conditions. In T1D, microbiome-oriented strategies are most plausibly positioned as stage-aware or early-life adjuncts, whereas in LADA they may need to be tailored to phenotype, with stronger emphasis on metabolic context in addition to autoimmune activity. The field is likely to move forward most effectively when endpoints match stages, functions match interventions, and reproducibility is treated as a primary outcome rather than an afterthought (1, 3, 100–102).

## 14 Conclusions

Current evidence is consistent with a model in which the gut microbiome acts as a modifier of risk and disease dynamics in autoimmune diabetes, primarily through effects on intestinal barrier integrity and immunometabolic signalling, based mainly on prospective and mechanistic convergence, particularly in T1D; evidence in LADA remains less mature. The more reproducible signals currently converge on microbial outputs (especially SCFAs, tryptophan-derived indoles, and bile acid-dependent pathways) rather than on any single taxonomic signature, based mainly on prospective and mechanistic convergence rather than intervention-level proof. This functional convergence helps explain why microbial signatures often vary across cohorts while mechanistic themes recur.

At the same time, microbiome science does not yet justify a stand-alone, universally applicable microbiome prevention strategy for T1D and LADA. Any near-term translation should therefore remain disciplined and stage-aware: upstream, prioritising low-risk interventions that support fermentation and epithelial homeostasis; and downstream, after seroconversion, prioritising structured surveillance and immune-directed approaches with demonstrated effects on clinically meaningful endpoints. Future progress is likely to depend on stage-stratified, function-oriented studies that integrate metagenomics with metabolomics and immune phenotyping, and that test metabolite-targeted interventions against hard outcomes such as time to seroconversion, time to dysglycaemia, and preservation of C-peptide.
