# Supplementary material for: Gut microbiota and the early prevention window in type 1 diabetes and latent autoimmune diabetes in adults: a state-of-the-art narrative review on diet and metabolites
Source: Front Endocrinol (Lausanne). 2026 May 8;17:1837746. doi: 10.3389/fendo.2026.1837746 (PMC13194072; doi:10.3389/fendo.2026.1837746)
Supplement: Supplementary file 1 [file DataSheet1.docx]

Supplementary Material

# Supplementary Figures and Tables

## Supplementary Figures


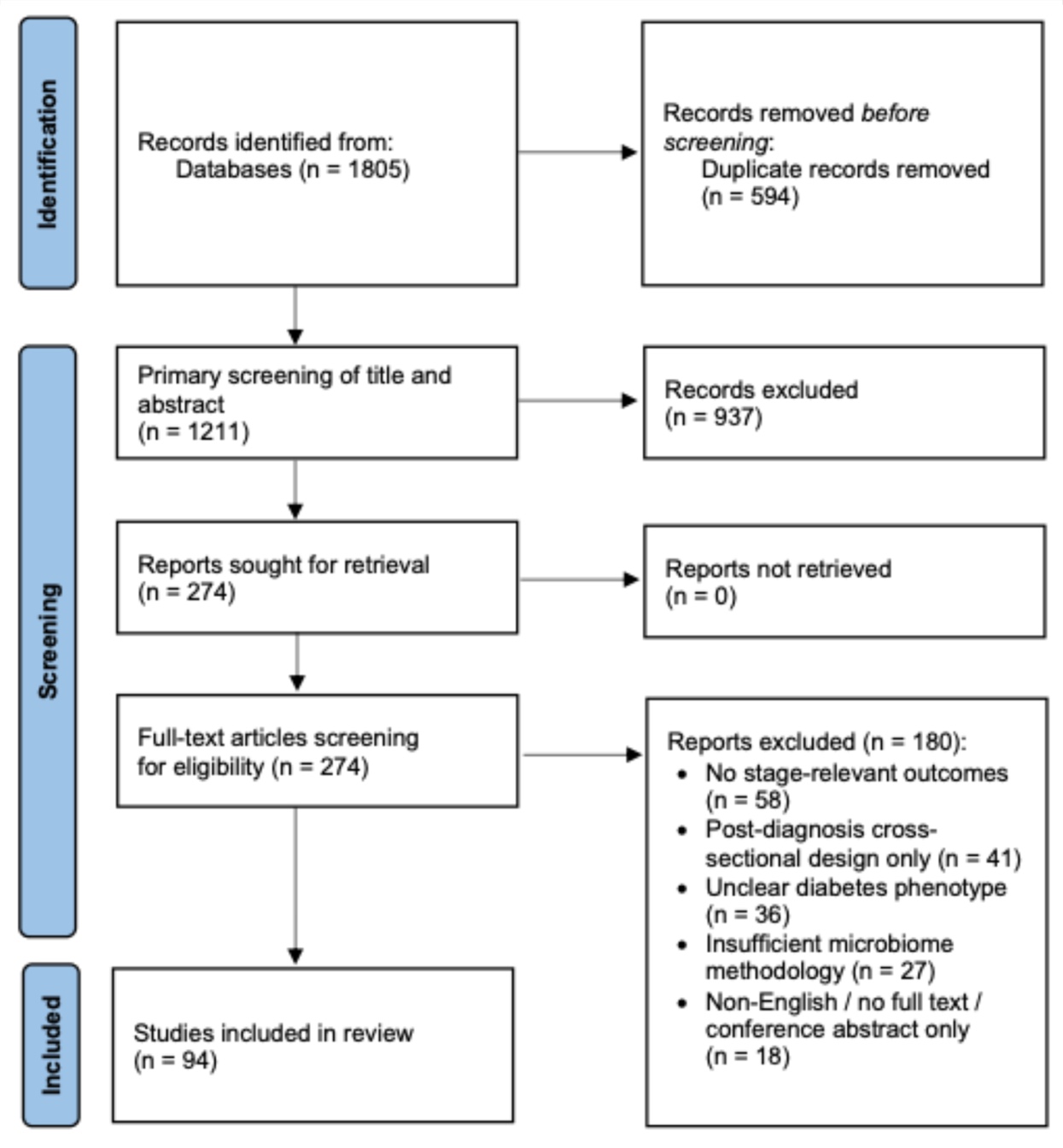


**Supplementary Figure 1.** Flow diagram of study selection process.

## Supplementary Tabels

**Supplementary Table S1.** Database-specific literature search strategy

| **PubMed** |
| --- |
| ((("Diabetes Mellitus, Type 1"[Mesh] OR "type 1 diabetes"[tiab] OR T1D[tiab] OR "autoimmune diabetes"[tiab] OR "islet autoimmunity"[tiab] OR "islet autoantibod*"[tiab] OR seroconversion[tiab] OR dysglyc*[tiab] OR "beta-cell"[tiab] OR "β-cell"[tiab] OR "latent autoimmune diabetes in adults"[tiab] OR LADA[tiab]) AND ("Gastrointestinal Microbiome"[Mesh] OR microbiom*[tiab] OR microbiota[tiab] OR "gut flora"[tiab] OR metagenom*[tiab] OR metabolom*[tiab] OR metatranscriptom*[tiab] OR "16S"[tiab] OR "shotgun metagenom*"[tiab])) AND (("intestinal barrier"[tiab] OR "gut barrier"[tiab] OR permeability[tiab] OR "tight junction*"[tiab] OR mucus[tiab] OR SCFA*[tiab] OR "short-chain fatty acid*"[tiab] OR butyrate[tiab] OR acetate[tiab] OR propionate[tiab] OR tryptophan[tiab] OR indole*[tiab] OR "aryl hydrocarbon receptor"[tiab] OR AhR[tiab] OR "bile acid*"[tiab] OR FXR[tiab] OR TGR5[tiab] OR Treg[tiab] OR "regulatory T cell*"[tiab] OR Th17[tiab] OR "innate immune*"[tiab]) OR (diet*[tiab] OR fiber[tiab] OR fibre[tiab] OR "resistant starch"[tiab] OR "ultra-processed"[tiab] OR antibiotic*[tiab] OR probiotic*[tiab] OR prebiotic*[tiab] OR synbiotic*[tiab] OR postbiotic*[tiab] OR "early life"[tiab] OR infancy[tiab] OR childhood[tiab] OR pregnancy[tiab] OR perinatal[tiab]))) |
| **Web of Science Core Collection** |
| TS=(("type 1 diabetes" OR T1D OR "autoimmune diabetes" OR "islet autoimmun*" OR "islet autoantibod*" OR seroconversion OR dysglyc* OR "beta cell*" OR "β-cell*" OR "latent autoimmune diabetes in adults" OR LADA) AND (microbiom* OR microbiota OR "gut flora" OR metagenom* OR metabolom* OR metatranscriptom* OR "16S" OR "shotgun metagenom*") AND (("intestinal barrier" OR "gut barrier" OR permeability OR "tight junction*" OR mucus OR SCFA* OR "short-chain fatty acid*" OR butyrate OR acetate OR propionate OR tryptophan OR indole* OR "aryl hydrocarbon receptor" OR AhR OR "bile acid*" OR FXR OR TGR5 OR Treg OR "regulatory T cell*" OR Th17 OR "innate immune*") OR (diet* OR fiber OR fibre OR "resistant starch" OR "ultra-processed" OR antibiotic* OR probiotic* OR prebiotic* OR synbiotic* OR postbiotic* OR "early life" OR infancy OR childhood OR pregnancy OR perinatal))) |
| **SCOPUS** |
| TITLE-ABS-KEY (("type 1 diabetes" OR T1D OR "autoimmune diabetes" OR "islet autoimmunity" OR "islet autoantibod*" OR seroconversion OR dysglyc* OR "beta-cell" OR "β-cell" OR "latent autoimmune diabetes in adults" OR LADA) AND (microbiom* OR microbiota OR "gut flora" OR metagenom* OR metabolom* OR metatranscriptom* OR "16S" OR "shotgun metagenom*") AND (("intestinal barrier" OR "gut barrier" OR permeability OR "tight junction*" OR mucus OR SCFA* OR "short-chain fatty acid*" OR butyrate OR acetate OR propionate OR tryptophan OR indole* OR "aryl hydrocarbon receptor" OR AhR OR "bile acid*" OR FXR OR TGR5 OR Treg OR "regulatory T cell*" OR Th17 OR "innate immune*") OR (diet* OR fiber OR fibre OR "resistant starch" OR "ultra-processed" OR antibiotic* OR probiotic* OR prebiotic* OR synbiotic* OR postbiotic* OR "early life" OR infancy OR childhood OR pregnancy OR perinatal))) |
| **Manual reference screening** |
| Reference lists of relevant systematic reviews, consensus statements, and landmark primary studies were screened manually to identify additional studies that were incompletely indexed or not optimally retrieved by database syntax, especially prospective birth/childhood cohorts, LADA cohorts, and mechanistically informative metabolite/barrier studies |

**Supplementary Table S2.** Key studies underpinning the stage-aware, function-first synthesis of gut microbiome research in autoimmune diabetes

| **Study** | **Population / phenotype** | **Stage of disease** | **Design** | **Microbiome platform** | **Functional axis** | **Main finding** | **Key limitation** | **Role in narrative weighting** |
| --- | --- | --- | --- | --- | --- | --- | --- | --- |
| Vatanen et al., 2018 [1] | Genetically at-risk children; early-onset T1D trajectory | Pre-seroconversion to preclinical progression | Prospective longitudinal cohort | Shotgun metagenomics | Function over taxonomy; community pathways | Associations with islet autoimmunity/progression were more coherent at the level of microbial function than single taxa | Observational; residual confounding; cohort/platform dependence | Primary prospective human anchor for the function-first argument |
| Zhang et al., 2022 [2] | Infants at risk for T1D | Stage 0–1 (seroconversion window) | Prospective infant microbiome study | Metagenome-assembled genomes / shotgun-based infant gut profiling | Early-life microbial maturation; seroconversion biology | Islet autoantibody seroconversion associated with infant gut metagenomic features | Needs replication; complex infant-exposure confounding | Supportive prospective signal for very-early-life staging |
| Uusitalo et al., 2016 [3] | Genetically high-risk infants | Infancy / pre-seroconversion | Prospective observational cohort | N/A (exposure study; no direct microbiome profiling) | Timing of probiotic exposure; early-life window | Very early probiotic exposure associated with lower risk of islet autoimmunity in selected high-risk children | Confounding by indication; not causal; no strain-level mechanistic proof | Timing-sensitive observational signal; hypothesis-strengthening, not practice-changing |
| Beyerlein et al., 2015 [4] | Genetically susceptible children | Early childhood / stage 0–1 | Prospective dietary exposure study | N/A (dietary exposure study) | Fibre as fermentation substrate; SCFA-related rationale | Higher soluble fibre intake associated with lower risk of islet autoimmunity | Indirect microbiome inference; not a direct functional readout study | Diet-to-function translational support for fibre/fermentation framing |
| Krischer et al., 2022 [5] | High-risk relatives/children in TEDDY | Initiation and progression staging | Prospective cohort | N/A (stage framework / predictors study) | Stage-aware natural history | Predictors of initiation and progression differ, reinforcing stage-specific interpretation | Not microbiome-specific | Stage-structure anchor for the review’s staged T1D logic |
| Mariño et al., 2017 [6] | NOD mice / preclinical autoimmune diabetes | Initiation / early progression | Preclinical mechanistic intervention | N/A or targeted metabolite/diet manipulation with immune phenotyping | SCFAs; Treg/immune tolerance | Microbial metabolites reduced autoimmune T-cell frequency and protected against diabetes | Preclinical model; translation to humans indirect | Primary mechanistic SCFA anchor |
| Bell et al., 2022 [7] | Adults with established T1D | Stage 3 / established disease | Small human mechanistic intervention | Microbiota profiling + immune modulation readouts | Metabolite-based supplementation; microbiota–immune interaction | Metabolite-based dietary supplementation associated with microbiota and immune modulation in human T1D | Small sample; surrogate endpoints; not prevention-oriented | Human proof-of-concept for metabolite-oriented translation |
| Yuan et al., 2022 [8] | Children with new-onset T1D | Stage 3 / post-diagnosis | Case-control multi-omics | Gut microbiota + metabolomic profiling | Functional/metabolic outputs | Functional and metabolic alterations reported in new-onset pediatric T1D | Cross-sectional; reverse causation after diagnosis | Supportive cross-sectional functional signal, weighted below pre-seroconversion cohorts |
| Fang et al., 2021 [9] | Adults with LADA | Established adult autoimmune diabetes | Case-control multi-omics | Gut microbiota + fecal/circulating metabolomics | Immunometabolic LADA framing; SCFA-related functional attenuation | Distinct microbiota and metabolomic features associated with autoantibody status, glucose metabolism, and islet function; recurrent signal of reduced SCFA-related capacity | Cross-sectional; medication/adiposity/diet confounding; replication needed | Primary LADA human anchor, but still hypothesis-generating relative to staged T1D |
| Poulsen et al., 2024 [10] | Adults with LADA | Established adult autoimmune diabetes | Cross-sectional characterization study | Bacterial + viral microbiota profiling | Broader ecosystem characterization in LADA | Extends LADA signal beyond bacterial-only framing | Exploratory; cross-sectional; still immature evidence base | Supportive LADA replication/extension study |
| Herold et al., 2019 [11] | Relatives at risk for T1D | Post-seroconversion / secondary prevention | Randomized trial | N/A (non-microbiome translational anchor) | Stage-specific downstream intervention | Teplizumab delayed progression to clinical T1D | Not microbiome-directed | Downstream translational comparator, showing that after seroconversion the strongest evidence comes from immune-directed therapy |

*Note: “Role in narrative weighting” reflects the review’s stage-aware, function-first synthesis framework, with greater weight assigned to prospective sampling, stage-relevant phenotyping, convergent mechanistic evidence, and replication or functional consistency across cohorts.*

*Abbreviations: LADA, latent autoimmune diabetes in adults; N/A, not applicable; NOD, non-obese diabetic; SCFAs, short-chain fatty acids; T1D, type 1 diabetes; TEDDY, The Environmental Determinants of Diabetes in the Young; TrialNet, Type 1 Diabetes TrialNet; Treg, regulatory T cells.*

References:

1. Vatanen T, Franzosa EA, Schwager R, Tripathi S, Arthur TD, Vehik K, Lernmark Å, Hagopian WA, Rewers MJ, She JX, Toppari J, Ziegler AG, Akolkar B, Krischer JP, Stewart CJ, Ajami NJ, Petrosino JF, Gevers D, Lähdesmäki H, Vlamakis H, Huttenhower C, Xavier RJ. The human gut microbiome in early-onset type 1 diabetes from the TEDDY study. Nature. 2018;562(7728):589-594. doi:10.1038/s41586-018-0620-2.
2. Zhang L, Jonscher KR, Zhang Z, Xiong Y, Mueller RS, Friedman JE, Pan C. Islet autoantibody seroconversion in type-1 diabetes is associated with metagenome-assembled genomes in infant gut microbiomes. Nat Commun. 2022;13(1):3551. doi:10.1038/s41467-022-31227-1.
3. Uusitalo U, Liu X, Yang J, Aronsson CA, Hummel S, Butterworth M, Lernmark Å, Rewers M, Hagopian W, She JX, Simell O, Toppari J, Ziegler AG, Akolkar B, Krischer J, Norris JM, Virtanen SM; TEDDY Study Group. Association of Early Exposure of Probiotics and Islet Autoimmunity in the TEDDY Study. JAMA Pediatr. 2016;170(1):20-28. doi:10.1001/jamapediatrics.2015.2757.
4. Beyerlein A, Liu X, Uusitalo UM, Harsunen M, Norris JM, Foterek K, Virtanen SM, Rewers MJ, She JX, Simell O, Lernmark Å, Hagopian W, Akolkar B, Ziegler AG, Krischer JP, Hummel S; TEDDY Study Group. Dietary intake of soluble fiber and risk of islet autoimmunity by 5 y of age: results from the TEDDY study. Am J Clin Nutr. 2015;102(2):345-352. doi:10.3945/ajcn.115.108159.
5. Krischer JP, Liu X, Lernmark Å, Hagopian WA, Rewers MJ, She JX, Toppari J, Ziegler AG, Akolkar B; TEDDY Study Group. Predictors of the Initiation of Islet Autoimmunity and Progression to Multiple Autoantibodies and Clinical Diabetes: The TEDDY Study. Diabetes Care. 2022;45(10):2271-2281. doi:10.2337/dc21-2612.
6. Mariño E, Richards JL, McLeod KH, Stanley D, Yap YA, Knight J, McKenzie C, Kranich J, Oliveira AC, Rossello FJ, Krishnamurthy B, Nefzger CM, Macia L, Thorburn A, Baxter AG, Morahan G, Wong LH, Polo JM, Moore RJ, Lockett TJ, Clarke JM, Topping DL, Harrison LC, Mackay CR. Gut microbial metabolites limit the frequency of autoimmune T cells and protect against type 1 diabetes. Nat Immunol. 2017;18(5):552-562. doi:10.1038/ni.3713.
7. Bell KJ, Saad S, Tillett BJ, McGuire HM, Bordbar S, Yap YA, Nguyen LT, Wilkins MR, Corley S, Brodie S, Duong S, Wright CJ, Twigg S, de St Groth BF, Harrison LC, Mackay CR, Gurzov EN, Hamilton-Williams EE, Mariño E. Metabolite-based dietary supplementation in human type 1 diabetes is associated with microbiota and immune modulation. Microbiome. 2022;10(1):9. doi:10.1186/s40168-021-01193-9.
8. Yuan X, Wang R, Han B, Sun C, Chen R, Wei H, Chen L, Du H, Li G, Yang Y, Chen X, Cui L, Xu Z, Fu J, Wu J, Gu W, Chen Z, Fang X, Yang H, Su Z, Wu J, Li Q, Zhang M, Zhou Y, Zhang L, Ji G, Luo F. Functional and metabolic alterations of gut microbiota in children with new-onset type 1 diabetes. Nat Commun. 2022;13(1):6356. doi:10.1038/s41467-022-33656-4.
9. Fang Y, Zhang C, Shi H, Wei W, Shang J, Zheng R, Yu L, Wang P, Yang J, Deng X, Zhang Y, Tang S, Shi X, Liu Y, Yang H, Yuan Q, Zhai R, Yuan H. Characteristics of the Gut Microbiota and Metabolism in Patients With Latent Autoimmune Diabetes in Adults: A Case-Control Study. Diabetes Care. 2021;44(12):2738-2746. doi:10.2337/dc20-2975.
10. Poulsen CS, Hesse D, Fernandes GR, et al. Characterization of the Gut Bacterial and Viral Microbiota in Latent Autoimmune Diabetes in Adults. Sci Rep. 2024;14(1):8315. doi:10.1038/s41598-024-58985-w.
11. Herold KC, Bundy BN, Long SA, Bluestone JA, DiMeglio LA, Dufort MJ, Gitelman SE, Gottlieb PA, Krischer JP, Linsley PS, Marks JB, Moore W, Moran A, Rodriguez H, Russell WE, Schatz D, Skyler JS, Tsalikian E, Wherrett DK, Ziegler AG, Greenbaum CJ; Type 1 Diabetes TrialNet Study Group. An Anti-CD3 Antibody, Teplizumab, in Relatives at Risk for Type 1 Diabetes. N Engl J Med. 2019;381(7):603-613. doi:10.1056/NEJMoa1902226.
